# Exercise in Pregnancy: The Impact of an Intervention Program in the Duration of Labor and Mode of Delivery

**DOI:** 10.1055/s-0038-1675613

**Published:** 2018-11-14

**Authors:** Cátia Liliana Martins Ferreira, Cláudia Maria Lopes Guerra, Ana Isabel Teixeira Jesus Silva, Helena Rafaela Vieira do Rosário, Maria Beatriz Ferreira Leite de Oliveira Pereira

**Affiliations:** 1 Gynecology and Obstetrics Service, Hospital Senhora da Oliveira, Guimarães, Portugal; 2 Institute of Education, University of Minho, Minho, Portugal; 3 Research Centre in Child Studies, University of Minho, Minho, Portugal; 4 School of Nursing, University of Minho, Minho, Portugal

**Keywords:** exercise, pregnancy, mode of delivery, duration of labor, labor induction, exercício, gravidez, via de parto, duração do trabalho de parto, indução do trabalho de parto

## Abstract

**Objective** To access the benefits or harms of an exercise program, based on the current American College of Obstetricians and Gynecologists guidelines, on the mode of delivery, duration and onset of labor.

**Methods** A study performed at the Hospital Senhora da Oliveira between October 2015 and February 2017. This was a quasi-experimental study involving 255 women divided into two groups: an intervention group engaged in a controlled and supervised exercise program during pregnancy (*n* = 99), and a control group that did not participate in the exercise program (*n* = 156). Data were collected in two stages: during the 1^st^ trimester biochemical screening (before the beginning of the program), through a written questionnaire, and after delivery, from the medical files of the patients. The significance level in the present study was 5% (*p* = 0.05).

**Results** The control group had higher odds of induced labor (odds ratio [OR] 2.71; 95% confidence interval [CI]: 1.42–5.17; *p* = 0.003), when compared with women who underwent the intervention. No differences were found between the groups in instrumental vaginal deliveries, cesarean rate, time until the beginning of the active phase, duration of the active phase, and duration of the second stage of labor.

**Conclusion** The implementation of a controlled and supervised exercise program in pregnancy was associated with significantly lower odds of induced deliveries.

## Abstract (pt)

**Resumo:**

**Objetivo** Avaliar o efeito de um programa de exercícios na gravidez (baseado nas recomendações do American College of Obstetricians and Gynecologists) na via, duração e início do trabalho de parto.

**Métodos** Estudo realizado no Hospital Senhora da Oliveira entre outubro de 2015 e fevereiro de 2017. Trata-se de um estudo quasi-experimental com 255 grávidas, divididas em dois grupos: grupo de intervenção, constituído por mulheres que participaram de um programa controlado e supervisionado de exercícios físicos (*n* = 99), e um grupo de controle, formado por grávidas que não participaram do programa de exercícios (*n* = 156). Os dados foram colhidos em dois momentos: durante o rastreio bioquímico do primeiro trimestre (antes do início do programa), através de um questionário escrito, e após o parto, através da consulta do processo clínico da paciente. O nível de significância estatística adotado neste projeto foi de 5% (*p* = 0,05).

**Resultados** O grupo controle apresentou maior probabilidade de indução do trabalho de parto (razão de chances [RC] 2,71; intervalo de confiança [IC] 95%: 1,42–5,17; *p* = 0,003) quando comparado com o grupo de intervenção. Não foram encontradas diferenças estatisticamente significativas entre os dois grupos em relação à via de parto, tempo até início da fase ativa, duração da fase ativa e duração do segundo estadio do trabalho de parto.

**Conclusão** A implementação de um programa de exercícios controlado e supervisionado na gravidez foi associada a uma probabilidade significativamente menor de indução do trabalho de parto.

## Introduction

Pregnancy is an ideal time for behavior modification and for adopting a healthy lifestyle because of the increased motivation and frequent access to medical supervision.^1^ According to the Committee Opinion number 650 of the American College of Obstetricians and Gynecologists (ACOG), women with uncomplicated pregnancies should be encouraged to engage in aerobic and strength conditioning exercise before, during and after pregnancy.^1^ An exercise program that leads to an eventual goal of moderate intensity exercise for at least 20 to 30 minutes per day on most or all days of the week should be developed.^1^ The available evidence indicates that the most favorable type of exercise intervention, at least regarding maternal health outcomes, is a combination of aerobic and resistance exercise.^2^ These physical activity guidelines were developed due to accumulating evidence that exercise is beneficial for both the mother and the fetus during pregnancy.^3^ Regular physical activity during pregnancy improves or maintains physical fitness, helps with weight management, reduces the risk of gestational diabetes in obese women, and enhances the psychological well-being.^1^ Contrary to these recommendations and regulations, statistics show that the majority of pregnant women remains sedentary or insufficiently active. Moreover, many women limit their physical activity during pregnancy.^4^

The beneficial effects of physical activity during pregnancy for the mother and the offspring have been reported by several studies, but there are conflicting results concerning the possible effect of physical activity on the course of labor and on the risk of cesarean delivery.^5^ Scientific evidence from experimental studies on the influence of exercise on the type of delivery is not consensual, and more studies in this field are needed.^6^ While there are studies that show that regular exercise in pregnancy decreases the risk of cesarean delivery,^5^ ^6^ ^7^ ^8^ ^9^ ^10^ ^11^ ^12^ ^13^ ^14^ ^15^ other studies show controversial results^16^ ^17^ ^18^ ^19^ or have failed to prove this association.^2^ ^3^ ^20^ ^21^ ^22^ ^23^ ^24^ ^25^ In relation to instrumental vaginal deliveries, the situation is similar: some investigations show an association between physical exercise and a lower rate of instrumental deliveries,^6^ ^11^ while others do not report this effect.^8^ ^10^ ^13^ ^14^ ^20^ ^23^

Physical fitness influences the course of labor mainly because exercise induces several metabolic and hormonal changes that may affect uterine contractility and endurance.^26^ Some scientific evidence suggests that regular exercise during pregnancy may be associated with shorter labor duration^26^ due to a shorter duration of the first stage,^20^ ^25^ ^27^ but other authors do not show any difference between regular exercise and the absence of exercise during pregnancy.^2^ ^10^ ^16^ ^17^ ^22^ ^23^ Furthermore, one recent study reported a longer first stage of labor in the exercise group.^5^ For the same reason, the onset of labor could be different between the sedentary and the exercise groups, with a higher incidence of spontaneous labor (and consequently less use of induction methods) in the exercise group.^9^ However, the majority of researches do not find higher induction rates in sedentary women.^8^ ^18^ ^20^ ^21^ In addition, some studies report differences between the two groups (with and without physical activity during pregnancy) regarding the duration of gestation after reaching term. Once again, the scientific evidence is not consensual: some authors support a possible association between physical activity and shorter duration of pregnancy at term,^9^ ^17^ whereas others do not.^8^ ^10^ ^20^ ^21^ ^22^ ^28^ ^29^

Until now, the benefits or harms of an exercise program on labor outcomes are not fully understood. Therefore, we aim to analyze the impact of an intervention program based on the current ACOG guidelines on labor outcomes, namely: Bishop score at admission, frequency of premature rupture of membranes, onset of labor (spontaneous versus induced), time until the beginning of the active phase, duration of the active phase, duration of the second stage of labor, use of epidural analgesia, need for episiotomy, frequency of third and fourth grade perineal lacerations, as well as frequency and indications of cesarean section (CS) and instrumental deliveries.

## Methods

### Study Design

The present study is a quasi-experimental study (registration number NCT03045237), which comprised an intervention group (with a physical activity program) and a control group (standard care). This is a secondary analysis, and the primary outcomes of the present study (gestational weight gain, postpartum depressive symptoms, and newborn weight and length) are waiting for publication.

### Participants

**Fig. 1:**
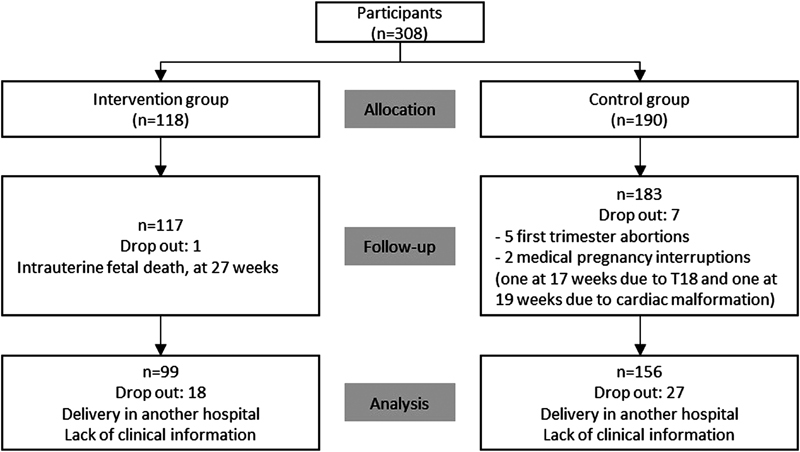
Consort flow chart of study population.

During the 1^st^ trimester biochemical screening in the Hospital Senhora da Oliveira (between the 8^th^ and 10^th^ weeks of gestation), women were invited to participate in the present study and to complete a written questionnaire. The Hospital Senhora da Oliveira is a differentiated perinatal support center, which attends low and high-risk pregnancies, following institutional clinical protocols for induction of labor and cesarean indications. Participants who wanted to perform the physical exercise program were included in the intervention group after the 12^th^ week, and the others formed the control group, which followed the standard care procedures provided by health professionals in Portugal. The control group maintained its usual routine and was not instructed to stop practicing physical exercise, if they did it routinely. A total of 99 women were included in the intervention group and 156 in the control group, between October 2015 and April 2017. Fig. 1 represents the flow of participants through the study. Inclusion criteria included women > 18 years old, with no medical or obstetrical contraindications to practice exercise (according to the ACOG Committee Opinion number 650).^1^

Moreover, it was mandatory that women attended at least ten classes of the program. Exclusion criteria included any type of absolute or relative contraindication to exercise suggested by the ACOG: hemodynamically significant heart disease, restrictive lung disease, incompetent cervix or cerclage, multiple gestation at risk of premature labor, persistent second or third trimester bleeding, placenta previa after 26 weeks of gestation, premature labor risk during the current pregnancy, ruptured membranes, preeclampsia or pregnancy induced hypertension, severe anemia (Hb < 7.0 mg/dL), unevaluated maternal cardiac arrhythmia, chronic bronchitis, poorly controlled type I diabetes, extreme morbid obesity, extreme underweight (body mass index [BMI] < 12 kg/m^2^), history of extremely sedentary lifestyle, intrauterine growth restriction in the current pregnancy, poorly controlled hypertension, orthopedic limitations, poorly controlled seizure disorder, poorly controlled thyroid disease, and heavy smokers (≥20 cigarettes/day).^1^ To be included in the study, the pregnant women had to present a document written by their assistant doctor attesting that they did not have none of these contraindications. Additional exclusion criteria included: women that delivered in other hospitals, lack of medical follow-up throughout the pregnancy, 1^st^ trimester abortions, medical pregnancy interruptions, and intrauterine fetal deaths.

### Intervention Program

The present study is part of a larger project called “Sporty Bellies.” The intervention program is a partnership between the University of Minho, the Hospital Senhora da Oliveira and the City Hall of Guimarães, Portugal. Its main goal is to promote healthy lifestyles in pregnancy, including regular physical exercise and a healthy and balanced diet. It comprised 10 training lessons (2 hours each) for teachers from the City hall, developed with the researchers, and their intervention with pregnant women. Besides the exercise program developed with trained teachers, the participants attended some theoretical sessions about healthy eating habits, breastfeeding, postpartum exercise, newborn care, and about the development of children during the 1^st^ year of life.

Women of the intervention group started the training program between the 12^th^ and 15^th^ weeks of gestation, until the end of the pregnancy. There were three classes per week, one of which was developed in an aquatic environment. The classes had a duration of between 45 and 50 minutes and were planned according to the recommendations of the ACOG.^1^ The classes consisted of: warm up (7 to 8 minutes), a fundamental part formed by aerobic, strength, coordination and flexibility exercises, performed in circuits and in stations (30 minutes), and return to rest (10 minutes). The physical program included pelvic musculature strengthening exercises. The activity performed was of moderate to vigorous intensity and, in order to measure exertion, the “talk test” was used, whose principle is that as long as women can carry out a conversation while exercising, she is likely not overexerting herself.^1^

### Outcome Measures

Data were collected in two stages: during the 1^st^ trimester biochemical screening (before the beginning of the program), and after delivery. Before the beginning of the program, the participants answered a questionnaire about the following variables: age, sociodemographic profile, smoking habits, physical activity, and weight and height prior to the pregnancy. After delivery, the following data were collected from the medical files of the patients: premature rupture of membranes, Bishop score at admission, onset of labor (spontaneous versus induced), time until the beginning of the active phase, duration of the active phase, duration of the second stage of labor, use of epidural analgesia, performance of episiotomy, frequency of third and fourth grade perineal lacerations, type of delivery, indications for cesarean and vaginal instrumental delivery, duration of the gestation, as well as the medical and obstetric history of the mother. The number of classes attended was controlled by the instructors during the classes through an attendance sheet. The sociodemographic profile was determined using the Graffar index. The prepregnancy BMI was calculated from self-reported prepregnancy weight and height. Prepregnancy physical activity was measured with the Pregnancy Physical Activity Questionnaire (P-PAQ), which consists of 32 questions grouped into different types of activity: household/caregiving, occupational, sports/exercise, transportation, and inactivity. The participants estimate the time spent in each activity (none, no less than 30 minutes per day, between 30 minutes and 1 hour per day, between 2 hours and 3 hours per day, and 3 hours or more per day). The activities are categorized by intensity as sedentary (< 1.5 metabolic equivalent of tasks [METs]), light (1.5–3.0 METs), moderate (3.1– 6.0 METs), or vigorous (> 6.0 METs). The duration of time spent in each activity is multiplied by its intensity to arrive at a measure of average weekly energy expenditure (MET hours per week [h/week]) attributable to each activity.^28^

The Bishop score was determined through cervix evaluation (dilation in centimeters, effacement as a percentage, consistency, position, and fetal situation).

The time until the active phase of labor was defined as the time elapsed between the time of admission of the patient and the presentation of 6 cm of dilatation. The duration of the active phase was defined as the time elapsed between the presentation of 6 cm of dilatation and the presentation of complete dilatation. The second stage was considered as the time elapsed between the moment when the cervix is fully dilated until the moment the baby is born.^29^ Instrumental Vaginal Deliveries Included only Vacuum Deliveries.

Third and fourth grade perineal lacerations were defined when the external and/or the internal anal sphincter were involved. Episiotomy was performed selectively. Epidural analgesia was performed according to the desire of the patient.

### Statistical Analysis

For sample size estimation, the variable of interest physical activity intensity in METs h/week was used. In Portugal, the mean (standard deviation [SD]) physical activity in pregnant women is 210.348 (116.753) METs h/week.^30^ To detect a 20% difference in physical activity between the groups in the third trimester (increasing average physical activity in intervention group by ∼ 42 METs h/week), with type I and II errors of 5% and 20%, it was found an effect size of 0.36. A sample of 244 pregnant women (122 in each group) was calculated at the second stage of data collection (after delivery). Assuming a dropout of 30%, it was proposed an initial sample size of 318 pregnant women (159 per group.)

Descriptive statistics are presented as absolute and relative frequencies, mean and SD and median and interquartile range, as appropriate. Group comparisons were assessed by the chi-squared test and the Fisher test for categorical variables, and by the Student t-test or the Mann–Whitney test for independent samples for continuous variables.

Logistic regression analyses were performed for delivery outcomes reporting adjusted odds ratio (OR) and 95% confidence interval (CI), adjusting for potential confounders such as age, prepregnancy BMI, smoking (yes/no), physical activity prior to gestation (METs h/week), socioeconomic status (category I and II/category III-V from the Graffar index), parity (nulliparous/multiparous), and previous cesarean (yes/no). In addition, in the variables “time until the active phase,” “length of the active phase” and “duration of the second stage of labor”, an additional adjustment was done for the onset of labor (induction/no induction).

The data analysis was performed using IBM SPSS Statistics for Windows, version 25.0 (IBM Corp., Armonk, NY, USA) and the level of significance was set at *p* < 0.05.

### Ethical Considerations

Prior to the participation in the study, all of the participants were informed about its purpose and a written informed consent was obtained, according to the ethical standards stated in the Declaration of Helsinki. The study was approved by the Subcommittee on Ethics for the Life Sciences and Health of the University of Minho (id: SECVS 086/2015) and by the Ethics Committee for Health of the Central Hospital (id: 056/2014).

## Results

Table 1 shows the baseline characteristics of the participants according to each group. The statistically significant differences are marked in bold. Women in the intervention group were older (32.0 ± 3.6 versus 30.7 ± 4.2; *p* = 0.018) and had a higher socioeconomic status (68 [68.7%] versus 77 [50.6%] in the category I-II of the Graffar index; *p* = 0.005). In addition, women from the control group had a higher proportion of smoking habit when compared with women who underwent the intervention (9.7% versus 3.0%; *p* = 0.040).

**Table 1:** Baseline characteristics of participants divided by intervention and control groups

Variable group	Control group (*n* = 156)	Intervention group (*n* = 99)	*p*-value
Sociodemografic factors			
Age, years old (mean [SD])	30.7 (4.2)	32.0 (3.6)	**0.018**
Prepregnancy BMI, kg/m2 (mean [SD])	24.2 (4.2)	23.5 (3.4)	0.151
BMI > 25 kg/m2 (*n* [%])	50 (32.1)	25 (25.3)	0.246
Socioeconomic status			
Category I-II Graffar index (*n* [%])	77 (50.6)	68 (68.7)	**0.005**
Category III-V Graffar index (*n* [%])	76 (49.4)	31 (31.3)	**0.005**
Smoking habit (*n* [%])	17 (9.6)	3 (3.0)	**0.044**
Prepregnancy physical activity, MET inh/week (mean [SD]) Obstetric history	187.3 (112.9)	163.0 (84.1)	0.121
Nulliparous (*n* [%])	87 (55.8)	67 (66.7)	0.058
Previous cesarean section (*n* [%])	22 (14.1)	12 (12.1)	0.650
Previous vaginal delivery (*n* [%]) Medical history	47 (30.1)	21 (21.2)	0.117
At least one medical problem (*n* [%])	24 (15.4)	19 (19.2)	0.429
Chronic hypertension (*n* [%])	6 (3.9)	0 (0)	0.084
Diabetes mellitus (*n* [%])	1 (0.6)	1 (1.0)	0.748
Thyroid disease (*n* [%])	5 (3.2)	4 (4.0)	0.732
Autoimune disease (*n* [%])	3 (1.9)	4 (4.0)	0.436
Other medical problem (*n* [%])	8 (5.2)	10 (10.1)	0.135

Abbreviations: BMI, body mass index; MET, metabolic equivalent of task; SD, standard deviation.

There were no statistically significant differences between the two groups regarding the number of deliveries after 41 weeks, the average Bishop score at admission, and premature rupture of membranes, as described in Table 2. The statistically significant differences are marked in bold. The Control group had a higher frequency of deliveries before 37 weeks (n [%], 10 [6.4] versus 1 [1.0]; *p* = 0.039), but after adjustment for confounding variables, this difference was no longer maintained (*p* = 0.073).

**Table 2:** Bishop score differences between the intervention and control groups regarding gestational age at delivery

Variable	Control group (*n* = 156)	Intervention group (*n* = 99)	*p*-value	OR	95% CI	*p*-value
Gestational age at delivery						
Delivery before 37 weeks (*n* [%])	10 (6.4)	1 (1.0)	**0.039**	7.05^c^	0.83–59.8	0.073
Delivery after 41 weeks (*n* [%])	13 (8.3)	4 (4.0)	0.180	2.45^c^	0.64–9.46	0.193
Bishop score at admission (mean [SD])	4.9 (2.1)	5.3 (2.1)	0.190	0.75^c^	0.21–1.31	0.515
Premature rupture of membranes (*n* [%])	52 (33.3)	32 (32.3)	0.867	1.12^c^	0.63–2.01	0.700
Induced labor (*n* [%])	53 (34.0)	20 (20.2)	**0.039**	**2.71** c	**1.42–5.17**	**0.003**
Indication for labor induction Gestation > 41 weeks (*n* [%])	18 (34.0)	4 (18.2)	0.246	1.69^c^	0.41–6.96	0.466
Premature rupture of membranes (*n* [%])	25 (47.2)	10 (50.0)	0.892	1.25^c^	0.39–4.03	0.710
Pregnancy related disease (*n* [%])	10 (18.9)	6 (30.0)	0.349	0.43^c^	0.11–1.75	0.242
Method of induction						
Misoprostol (*n* [%])	43 (27.6)	15 (15.2)	0.563	2.67^c^	1.32–5.43	0.730
Dose of misoprostol used, µg (mean [SD])	69.8 (56.1)	75.0 (42.3)	0.158	2.34^c^	1.23–4.82	0.186
Dinoprostone vaginal delivery system (*n* [%])	6 (3.8)	2 (2.0)	0.415			d
Oxytocin (*n* [%])	4 (2.6)	3 (3.0)	0.824			d

Abbreviations: CI, confidence interval; OR, odds ratio; SD, standard deviation.

c Adjusted for age, pre-pregnancy BMI, smoking habit, prepregnancy physical activity, socioeconomic status, parity, and previous cesarean.

d Adjusted analysis not performed due to the small number of cases.

Regarding the onset of labor, the control group had a higher rate of induced labor (n [%], 53 [34.0] versus 20 [20.2]; *p* = 0.039), even after adjusting for confounders. Being in the control group was associated with higher odds of an induced labor (OR 2.71; 95% CI.: 1.42–5.17; *p* = 0.003).

Regarding the time until the active phase, the duration of the active phase, and the duration of the second stage of labor, there were no differences among the control and the intervention group, and these results remained unchanged after adjusting for confounders. Similar results were observed in relation to the use of epidural analgesia (*p* = 0.110), performance of episiotomy (*p* = 0.450), breech presentations (*p* = 0.218), occipitoposterior varieties (*p* = 0.955), and third/fourth grade perineal lacerations (*p* = 0.082), as shown in Table 3.

**Table 3:** Labor outcomes for the control and intervention groups

Variable	Control group (*n* = 156)	Intervention group (*n* = 99)	*p*-value
Time until the active phase, hours (mean [SD])	8.7 (8.0)	7.8 (7.1)	0.241
Duration of the active phase, hours (mean [SD])	3.1 (2.1)	2.9 (2.0)	0.584
Duration of the 2^nd^ stage of labor, minutes (mean [SD])	33.4 (31)	35.8 (35.6)	0.701
Occipitoposterior varieties (*n* [%])	22 (33.8)	15 (33.3)	0.955
Breech presentations (*n* [%])	8 (5.1)	2 (2.0)	0.218
Epidural analgesia (*n* [%])	134 (97.1)	87 (92.6)	0.110
Episiotomy (*n* [%])	87 (66.4)	65 (72.2)	0.450
3^rd^ or 4^th^ grade perineal lacerations (*n* [%])	0 (0.0)	2 (2.2)	0.082
Cesarean delivery (*n* [%])	50 (32.1)	27 (27.3)	0.418
Failure to progress in labor (*n* [%])	20 (40.0)	7 (25.9)	0.217
Non-reassuring fetal state (*n* [%])	15 (30.0)	13 (48.1)	0.114
Abnormal fetal presentation (*n* [%])	9 (18.0)	2 (7.4)	0.311
Other indication (*n* [%])	6 (12.0)	5 (18.5)	0.503
Instrumental vaginal delivery (*n* [%])	24 (15.4)	20 (20.2)	0.321
Failure to progress in labor (*n* [%])	14 (58.3)	13 (65.0)	0.651
Non-reassuring fetal state (*n* [%])	9 (37.5)	7 (35.0)	0.864

Abbreviations: SD, standard deviation.

There were no differences between the groups regarding the rate of CSs or of instrumental vaginal deliveries. No differences were observed between the control and the intervention groups in relation to cesarean indications, and non-reassuring fetal state (n [%], 15 [30.0] versus 13 [48.1]; *p* = 0,039) and failure to progress in labor (n [%], 20 [40.0] versus 7 [25.9]; *p* = 0.039) were the main motives reported. The results were similar in relation to indications for vaginal instrumental delivery, in which cases failure to progress in labor were the main reason to proceed to a vacuum fetal extraction (n [%], 14 [58.3] in the control group versus 13 [65.0] in the intervention group; *p* = 0.651).

## Discussion

The main finding of the present study is that the intervention group had a lower rate of induced births compared with the control group. Belonging to the control group resulted in significantly higher odds of having an induced birth. These results are in line with Portela et al (2014),^9^ who suggest that women who practice exercise are likely to have a spontaneous vaginal delivery. However, it disagrees with some studies,^21^ including more recent investigations^20^ and meta-analyses,^8^ ^18^ in which there was no difference in the induction rates between the two groups. A possible explanation for this finding can be that physical activity induces some metabolic and hormonal changes that may affect uterine contractility, thus increasing the possibility of spontaneous onset of labor. Regarding the way of delivery, regular exercise in pregnancy was not associated with a lower cesarean rate, contrary to the results of two recent meta-analyses^7^ ^8^ and several studies.^5^ ^6^ ^9^ ^10^ ^11^ ^12^ ^13^ ^14^ ^15^ However, some works have reached similar conclusions and have not proved this association either.^2^ ^3^ ^16^ ^17^ ^18^ ^20^ ^21^ ^22^ ^23^ ^24^ ^25^ Several studies have results similar to ours in vacuum deliveries; no difference was found between regular exercise during pregnancy and a lower rate of instrumental vaginal deliveries,^10^ ^13^ ^14^ ^20^ ^23^ including a recent Cochrane revision from 2017.^8^ In relation to the duration of labor, there were no differences between the two groups, which is in line with that was reported by some authors,^2^ ^10^ ^16^ ^17^ ^22^ ^23^ ^31^ and in disagreement with others.^5^ ^20^ ^25^ ^26^ ^27^ The disparity in the results may be explained by distinct definitions of the stages of labor. In the present investigation, the active phase was considered the time elapsed between the presentation of 6 cm of dilatation and the presentation of complete dilatation, according to Zhang et al (2010).^29^ Other authors considered the first stage of labor as the time elapsed between the presentation of 3 or 4 cm of dilatation and full dilatation, and did not divide it into latent and active phases.^5^ ^26^ In addition, we do not have data about labor augmentation with oxytocin, which plays an important role in the duration of labor.

The differences in our results compared with other studies can be attributed to the variety of study design, type and intensity of the exercise and duration of the programs used in these studies. Possible explanations for the lack of effect of the exercise practice on labor duration and on the mode of delivery can be the following reasons: first of all, the number of classes required to be part of the intervention group may have been low (10 sessions), perhaps too low to influence the study outcomes. Second, another limitation was the lack of rigor in monitoring class attendance, making difficult to accurately determine the number of classes attended by each participant, to determine the effect of the amount of exercise in labor and delivery outcomes. Third, the control group was not instructed to not practice physical activity, so they may have practiced exercise outside the intervention program. Fourthly, the daily physical activity performed by women outside of the exercise program was not accessed in either group (intervention or control). We are aware that the non-randomization of the women among groups is another limitation of the present study. Those who chose to join the exercise group may be different from the patients of the control group in several ways, including their points of view and ideals about health, wellness and dietary habits. This could lead to a significant selection bias.

If, on the one hand, the present work failed to demonstrate the benefit of exercise in the mode of delivery and in the duration of labor, such as reported by several already mentioned recent works, on the other hand, the safety of physical activity in pregnancy was, once again, reaffirmed, since the intervention group did not show an increased rate of preterm delivery, which is in accordance with the majority of the authors.^3^ ^7^ ^8^ ^9^ ^10^ ^17^ ^18^ ^20^ ^21^ ^22^ ^32^ ^33^ ^34^ ^35^

The main strength of the present study is the prospective analysis, with several variables gathered and used as confounders. The number of previously sedentary healthy women who were enrolled in the project (*n* = 41) is an additional contribution of the present work. The general compliance with the exercise program was good for many reasons: the classes were held after 6 PM, allowing the women to exercise after work, the class plan was diversified in an attempt to capture the interest of the women, and the bonding process developed between the participants certainly potentiated class attendance.

## Conclusion

In the present study, the implementation of a controlled and supervised exercise program in pregnancy was associated with a lower rate of induced deliveries, which can be an additional incentive to regular physical activity during gestation. This work contributes to future researches that include physical activity during pregnancy, in order to improve maternal outcomes, including less induced deliveries.
